# 

**Published:** 1887-05

**Authors:** 


					﻿Entered at the Post Office, at Austin, Texas, as Second Class Mail Matter
Vol. it
MAY. iS3;.
No. II.
DANIEL'S
MEDICAL
JOURNAL
F. E. DANIEL., M. D., Editor and Publisher, AUSTIN, T?.'' A.C-
Aorihrrn Office. tS£7 FSHM-rt Street. I»l»Hade!phhk.
Ekoh Vox Bqeckmank, Stxam IJoof. anuJoh Pinter, Austin, Tax
				

